# Regulation of Neutrophil Functions by Hv1/VSOP Voltage-Gated Proton Channels

**DOI:** 10.3390/ijms22052620

**Published:** 2021-03-05

**Authors:** Yoshifumi Okochi, Yasushi Okamura

**Affiliations:** 1 Integrative Physiology, Graduate School of Medicine, Osaka University, 2-2 Yamada-oka, Suita 5650871, Osaka, Japan; yokamura@phys2.med.osaka-u.ac.jp; 2 Graduate School of Frontier Bioscience, Osaka University, 2-2 Yamada-oka, Suita 5650871, Osaka, Japan

**Keywords:** voltage-gated proton channel, phagocytes, neutrophils, ROS, NADPH oxidase, membrane potential, pH, Ca^2+^, degranulation, migration

## Abstract

The voltage-gated proton channel, Hv1, also termed VSOP, was discovered in 2006. It has long been suggested that proton transport through voltage-gated proton channels regulate reactive oxygen species (ROS) production in phagocytes by counteracting the charge imbalance caused by the activation of NADPH oxidase. Discovery of Hv1/VSOP not only confirmed this process in phagocytes, but also led to the elucidation of novel functions in phagocytes. The compensation of charge by Hv1/VSOP sustains ROS production and is also crucial for promoting Ca^2+^ influx at the plasma membrane. In addition, proton extrusion into neutrophil phagosomes by Hv1/VSOP is necessary to maintain neutral phagosomal pH for the effective killing of bacteria. Contrary to the function of Hv1/VSOP as a positive regulator for ROS generation, it has been revealed that Hv1/VSOP also acts to inhibit ROS production in neutrophils. Hv1/VSOP inhibits hypochlorous acid production by regulating degranulation, leading to reduced inflammation upon fungal infection, and suppresses the activation of extracellular signal-regulated kinase (ERK) signaling by inhibiting ROS production. Thus, Hv1/VSOP is a two-way player regulating ROS production. Here, we review the functions of Hv1/VSOP in neutrophils and discuss future perspectives.

## 1. Introduction

Voltage-gated proton channels are highly proton-selective channels. Proton currents were first recorded from snail neurons in 1982 [1], but their molecular basis remained unidentified for more than two decades. Later, voltage-gated proton currents were described in mammalian cells, such as rat alveolar epithelial cells [2] and human granulocytes [3,4]. Voltage-gated proton currents are now known to be present in many mammalian cells, including epithelial cells and immune cells [5].

Hv1/VSOP was discovered in a mouse cDNA database by a bioinformatics approach based on sequence similarity to the voltage sensor domain of *Ciona intestinalis* voltage-sensing phosphatase (Ci-VSP) [6,7]. Interestingly, the Hv1/VSOP protein has a voltage sensor domain, but lacks a pore domain, which all ion channels possess. It was, therefore, named VSOP (voltage-sensor domain-only protein). However, the name Hv1 is now more commonly used, following the nomenclature of other channels such as Na_v_ (voltage-gated sodium channel), K_v_ (voltage-gated potassium channel) and Ca_v_ (voltage-gated calcium channel). Hv1/VSOP is the only known voltage-gated proton channel, and no other isoforms have been identified. Hv1/VSOP has a transmembrane domain which consists of four helix segments that sense membrane potential. In addition to the transmembrane domain, Hv1/VSOP has a coiled-coil domain in its C-terminal cytoplasmic region that is necessary for dimerization and cooperative gating of the channel [8,9]. Proton permeation is mainly regulated by two factors, membrane potential and ΔpH (pH_out_ − pH_in_) [5,9]. Depolarized membrane potential induces Hv1/VSOP activation, and increased ΔpH facilitates proton permeation [5,10]. Hv1/VSOP is sensitive to external Zn^2+^ [10], which inhibits voltage-dependent S4 movement by interacting with several amino acids including two histidine residues [11].

Hv1/VSOP is expressed in immune cells [10,12,13,14], human sperm [15], and upper respiratory tract epithelial cells [16]; most of these cells (except for sperm) correspond to cells in which proton currents were previously recorded. Hv1/VSOP is expressed in almost all types of immune cells, including granulocytes [12,17,18], lymphocytes [13,14], macrophages [19,20], and dendritic cells [21]. Functions of Hv1/VSOP in neutrophils are summarized in Table 1.

Genetic variation including alternative splicing and single-nucleotide polymorphisms (SNPs) of human *HVCN1* (gene name of Hv1/VSOP) was found in two groups. One is from the analysis of this channel in B cells [31], and the other is from the trachea [16]. Two human Hv1/VSOP isoforms were expressed in B cells [31]. The shorter isoform that lacks the first 20 amino acids of this protein was enriched in B cells from patients with chronic lymphocytic leukemia (CLL) compared with the longer isoform most widely expressed in many cells [31]. The B lymphoma cell line overexpressing the short isoform exhibited enhanced B cell receptor-dependent signaling, increasing proliferation and migration, suggesting that it may contribute to the pathogenesis of B cell malignancy. Single-nucleotide polymorphisms (SNPs) in human *HVCN1* were accidentally found in the analysis of Hv1/VSOP in donor primary airway epithelia [16]. In addition, one *HVCN1* allele identified out of 95 human DNA samples was heterozygous for M91T mutation [16]. The M91T mutation caused reduced proton channel activity in heterologous expression systems and reduced acid secretion in primary airway cultures [16]. However, no clinical implication following this finding has been reported. No human patients having Hv1/VSOP deficiency have been described so far.

Functions of voltage-gated proton channels have been analyzed in granulocytes, especially eosinophils [32,33] and neutrophils [3,4,34]. This is because voltage-gated proton channels associate with superoxide-generating molecules, such as NADPH oxidase [34]. NADPH oxidase is a protein complex composed of membrane proteins and cytosolic proteins: the former are gp91 and p22, and the latter are p47, p67 and Rac [35]. When the membrane proteins interact with the cytosolic proteins, the oxidase becomes active. The active form of NADPH oxidase takes electrons from NADPH and releases protons inside cells. Then, electrons are transferred to oxygen outside cells to generate superoxide anions (O_2_^−^). The movement of electrons passing through the membrane via the active oxidase causes depolarization. Simultaneously, protons are released into the intracellular space, which could acidify the cytoplasm. Using a combination of a fluorescent voltage probe and a voltage-gated proton channel inhibitor, Cd^2+^, Henderson et al. showed that voltage-gated proton channels inhibit depolarization in neutrophils [34]. They showed that activation of NADPH oxidase by phorbol 12-myristate 13-acetate (PMA) induces neutrophil depolarization [34]. Inhibiting the voltage-gated proton channel increased the depolarization of PMA-stimulated neutrophils [34]. These results indicate that voltage-gated proton channels compensate for charge imbalances caused by the activation of NADPH oxidase.

Neutrophils normally circulate in blood, and these cells quickly migrate to sites of infection and then engulf and kill pathogens by releasing proteases and producing large amounts of reactive oxygen species (ROS) [36]. ROS production is initiated by the generation of O_2_^−^, which is rapidly converted to hydrogen peroxide (H_2_O_2_) by dismutation. H_2_O_2_ is converted into hydrogen radicals (HO^·^) and hypochlorous acid (HOCl) in the presence of Fe^2+^ and myeloperoxidase (MPO), respectively, both of which are strong oxidants capable of killing pathogens. MPO is released from granules by exocytosis [30], as described below.

## 2. Regulation of ROS Production by Hv1/VSOP in Neutrophils

### 2.1. Function of Hv1/VSOP on the Plasma Membrane

To confirm that Hv1/VSOP is the molecule responsible for charge compensation, the localization of Hv1/VSOP protein in several organs was investigated using specific antibodies [12,26]. Hv1/VSOP protein was detected mainly in the spleen, lung, bone marrow, peripheral blood cells, and neutrophils [12,26].

Hv1/VSOP-deficient mice were independently established by our, and other groups [12,14,26]. Using neutrophils, ROS were measured outside cells; Hv1/VSOP-deficient neutrophils exhibited less ROS production than wild-type neutrophils [12,26], indicating a contribution of Hv1/VSOP to ROS production.

**Figure 1:**
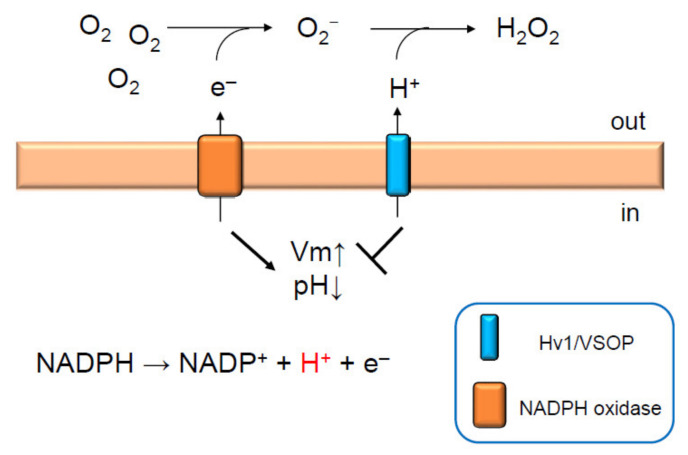
Hv1/VSOP maintains NADPH oxidase activity in neutrophils. Activation of NADPH oxidase depolarizes membrane potential. This is dampened by the activation of Hv1/VSOP, which compensates for charge imbalance [22]. Protons released into the cytoplasm upon NADPH oxidase activation are extruded by Hv1/VSOP outside the cell or into phagosomes to produce H_2_O_2_ and to maintain neutral phagosomal pH [22]. Thus, Hv1/VSOP has dual functions; inhibition of membrane depolarization and acidification of cytoplasm by extruding protons. Both lead to sustained ROS production by NADPH oxidase [12,22,26,28]. Arrow and T arrow indicate promotion and inhibition, respectively, for Vm and pH.

The activity of NADPH oxidase is inhibited by depolarization [37], which is induced by activation of the oxidase itself. As mentioned above, voltage-gated proton channels inhibit depolarization induced by NADPH oxidase activation [34]. Based on this evidence, a model was proposed: compensation of charge imbalance by voltage-gated proton channels is essential for sustaining NADPH oxidase activity [37] (Figure 1). To provide evidence supporting this model, membrane potential was measured by using a fluorescent probe, DiBAC_4_(3) [22]. The extent of membrane depolarization of Hv1/VSOP-deficient neutrophils was significantly greater than that of wild-type neutrophils when stimulated with PMA [22]. Excess depolarization was also observed in wild-type neutrophils when the activity of Hv1/VSOP was inhibited with Zn^2+^ [22]. These results indicate that Hv1/VSOP counteracts the charge imbalance induced by NADPH oxidase activation, which sustains the oxidase activity (Figure 1). During the oxidase activation, protons should accumulate in the cytoplasm of Hv1/VSOP-deficient neutrophils. Indeed, acidification was observed in these cells using BCECF, a pH-sensitive dye, under sodium-free extracellular conditions, which masks the function of the Na^+^/H^+^ exchanger, another regulator of cytoplasmic pH [22]. Acidification of the cytoplasm during phagocytosis in Hv1/VSOP-deficient neutrophils was also observed by two other groups [23,24]. These results indicate that Hv1/VSOP extrudes protons on the activation of NADPH oxidase. Preventing cytoplasm acidification via Hv1/VSOP is probably important to maintain ROS production in neutrophils, as previously suggested [5].

Hv1/VSOP is expressed in human neutrophils as well as mouse neutrophils. The contribution of Hv1/VSOP to ROS generation in human neutrophils was examined using a human leukemia cell line, PLB-985 [27]. Differentiated PLB-985 cells in the presence of dimethylformamide resemble neutrophils in morphology and function. Using neutrophil-like differentiated PLB-985 cells in combination with RNA interference, it was shown that human Hv1/VSOP maintains ROS production [27], as shown in mouse neutrophils.

### 2.2. Function of Hv1/VSOP on Phagosomes

Hv1/VSOP localizes on phagosomal membranes in neutrophils [12]; therefore, it is assumed that Hv1/VSOP regulates ROS production in phagosomes as well as on the plasma membrane. Phagosomes are sequestrated intracellular vesicles formed by phagocytosis, where pathogens are killed and digested by ROS. ROS within phagosomes were measured using ROS-sensitive fluorescent dye-conjugated zymosan particles [19], from the insoluble fraction of yeast. Phagosomal ROS were diminished in Hv1/VSOP-deficient neutrophils [19]. The amount of each subunit of NADPH oxidase recruited to phagosomes was not altered in Hv1/VSOP-deficient neutrophils [12], indicating that the reduced ROS production in phagosomes was not because of the number of active NADPH oxidase complexes, but because of reduced NADPH oxidase activity, as in the plasma membrane.

In parallel with ROS production, the pH in phagosomes drastically changes. Because cytotoxic proteins, such as elastase and cathepsin, released in phagosomes have their own optimal pH for activation, phagosomal pH is crucial for pathogen killing. Corresponding to the optimal pH of these proteins, the pH in phagosomes differs between neutrophils and macrophages. Phagosomal pH in macrophages is acidic [38], while their pH in neutrophils is neutral [39]. The difference in phagosomal pH between the two types of phagocytes has been explained by the amount of two proteins, NADPH oxidase and the proton pump, V-ATPase [39,40]. Because neutrophils are capable of producing large amounts of ROS, protons are consumed in the generation of H_2_O_2_ within phagosomes, thereby maintaining a neutral phagosomal pH. However, macrophages produce fewer ROS than neutrophils, resulting in the acidification of phagosomes. Inhibition of NADPH oxidase activity causes the acidification of phagosomes in neutrophils, whereas inhibiting V-ATPase activity causes alkalinization [39]. Moreover, V-ATPases do not accumulate efficiently on phagosomes during ROS generation [39]. These results indicate that the balance of NADPH oxidase and V-ATPase activities on phagosomes determine phagosomal pH. Because phagosomal ROS production was reduced in Hv1/VSOP-deficient neutrophils, it was speculated that phagosomal pH in Hv1/VSOP-deficient neutrophils is altered. Indeed, a large population of phagosomes is alkalinized in Hv1/VSOP-deficient neutrophils, whereas a small population of phagosomes is acidified [19]. Both populations were significantly higher than those of wild-type neutrophils. More detailed analysis showed that ROS production was inversely correlated with the accumulation of V-ATPase on phagosomes in the wild type [19]; the recruitment of V-ATPase onto phagosomes was diminished when large amounts of ROS were generated in the phagosomes. However, the inverse correlation observed in wild-type neutrophils was lost in Hv1/VSOP-deficient neutrophils [19]. Treatment with gramicidin, a monovalent cation-selective ionophore, restored the inverse correlation lost in Hv1/VSOP-deficient neutrophils [19]. Thus, Hv1/VSOP inhibits the recruitment of V-ATPase by maintaining high-level ROS production in phagosomes of neutrophils.

The function of Hv1/VSOP on phagosomes was also investigated in living zebrafish [28]. A significantly increased number of phagosomes was found in Hv1/VSOP-deficient neutrophils of zebrafish when bacteria were subcutaneously injected [28], indicating a defect in phagosomal function. Hv1/VSOP-deficient neutrophils isolated from zebrafish blood produced little ROS when stimulated with PMA [28]. These findings, together with the function of Hv1/VSOP in mouse neutrophil phagosomes, indicate that Hv1/VSOP in zebrafish probably eliminates bacteria by maintaining pH homeostasis and ROS production in phagosomes of neutrophils.

### 2.3. Impact of Hv1/VSOP Deficiency on Cell and Whole Animal Functions

Hv1/VSOP deficiency in mouse neutrophils lowers bacterial clearance in vitro [26], indicating that Hv1/VSOP-mediated maintenance of high level ROS production and pH homeostasis in phagosomes is necessary to effectively eliminate bacteria. In contrast to the in vitro study, Hv1/VSOP-deficient mice did not show any significant difference in bacterial elimination compared with wild-type mice [26]. This suggests the existence of compensatory mechanisms for the reduced bacterial clearance in Hv1/VSOP-deficient neutrophils in vivo.

## 3. Degranulation and Inflammation Regulated by Hv1/VSOP in Neutrophils

### 3.1. Degranulation Regulated by Hv1/VSOP

Degranulation is a key function in the killing and degradation of pathogens in neutrophils. Degranulation is tightly regulated by multiple steps because granules contain cytotoxic proteins that would damage host cells as well as pathogens. Neutrophils have four kinds of granules; secretory vesicles, tertiary (gelatinase) granules, secondary (specific) granules and primary (azurophilic) granules. Each granule is known to be released dependent on concentrations of Ca^2+^ [41,42,43] and GTP [44,45]: lower Ca^2+^ promotes the release of secretory vesicles, while higher Ca^2+^ induces primary granule release [41,42,43]. Likewise, GTP facilitates the hierarchical release of these granules [44,45].

**Figure 2:**
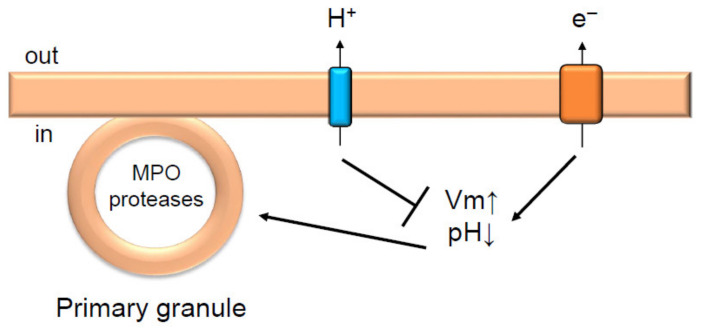
Hv1/VSOP inhibits degranulation in neutrophils. Hv1/VSOP inhibits degranulation of primary granules by regulating charge imbalance accompanied by NADPH oxidase activation [29]. Arrow and T arrow indicate promotion and inhibition, respectively, for Vm, pH and degranulation of primary granules. MPO is myeloperoxidase.

Hv1/VSOP inhibits the degranulation of primary granules in neutrophils [29] (Figure 2). In Hv1/VSOP-deficient neutrophils, excess MPO was released from cells when activated by PMA [29], leading to higher HOCl production compared with wild-type neutrophils [29], although Hv1/VSOP-deficient neutrophils produced less O_2_^−^ and H_2_O_2_ than wild-type neutrophils [12,22]. The inhibition of degranulation by Hv1/VSOP depends on NADPH oxidase activity; excess degranulation observed in Hv1/VSOP-deficient neutrophils was suppressed when the activity of the oxidase was inhibited by the NADPH oxidase inhibitor, diphenyleneiodonium (DPI) [29]. Additionally, Zn^2+^ mimicked the phenotype of Hv1/VSOP-deficient neutrophils in the wild type: Zn^2+^ induced excess degranulation in wild-type neutrophils stimulated with PMA [29]. These results indicate that Hv1/VSOP on the plasma membrane inhibits degranulation of primary granules. The inhibitory effect of Hv1/VSOP on degranulation appears specific to primary granules, because degranulation of secondary granules is not altered in Hv1/VSOP-deficient neutrophils [29].

As mentioned above, primary granules require a high intracellular level of Ca^2+^ for their degranulation [41,42,43]. However, Ca^2+^ entry via the plasma membrane is diminished in PMA-stimulated Hv1/VSOP-deficient neutrophils [22]. It is unlikely that Ca^2+^ is the key factor in excess degranulation observed in Hv1/VSOP-deficient neutrophils. Membrane depolarization in Hv1/VSOP-deficient neutrophils is more remarkable than in wild-type neutrophils when activated by PMA [22]; therefore, it was assumed that the charge imbalance causes excess degranulation. Potassium efflux is known to compensate for charge imbalance caused by NADPH oxidase activation, and charge imbalance can be cancelled by using the K^+^ permeable ionophore, valinomycin [40]. Valinomycin treatment of Hv1/VSOP-deficient neutrophils partially suppressed the excess degranulation [29]. This result suggests that Hv1/VSOP inhibits degranulation, in part through compensating the increase in positive charge caused by NADPH oxidase activation (Figure 2). The partial suppression of excess degranulation by valinomycin in Hv1/VSOP-deficient neutrophils raised the possibility that cytoplasm pH also affects the excess degranulation. Cytoplasmic pH in PMA-stimulated Hv1/VSOP-deficient neutrophils was lower than in wild-type neutrophils [22]; therefore, the lower cytoplasm pH might also facilitate the excess degranulation.

Molecular mechanisms of degranulation regulated by Hv1/VSOP are still unknown, but it is speculated that Hv1/VSOP may regulate the activity of Rac, a small GTPase. It is well known that Rac is essential for the activation of NADPH oxidase [46]. In neutrophils, Rac2 mainly contributes to ROS generation [46]. Rac2 also has a role in the specific degranulation of primary granules [47], suggesting that Hv1/VSOP inhibits degranulation by controlling Rac2. Several small GTPases, including Rac and K-Ras (as mentioned later), have a polybasic region formed by positively charged amino acids [48]. The polybasic region is necessary for tethering to the plasma membrane via electrostatic interaction with negatively charged phospholipids [48]. A striking finding is that the electrostatic interaction between small GTPases and phospholipids is affected by membrane potential [49]. Zhou et al. showed that membrane depolarization enhances nanoclustering of negatively charged phospholipids, facilitating nanoclustering of K-Ras on the plasma membrane [49]. They also showed that the polybasic region of K-Ras is important for nanoclustering upon depolarization [49]. These results indicate that the polybasic region of small GTPases is important for amplifying signals in association with membrane depolarization. Hv1/VSOP may inhibit nanoclustering of Rac2 by inhibiting depolarization, thereby suppressing excess degranulation of primary granules.

### 3.2. Inflammation Regulated by Hv1/VSOP

Excess secretion of cytotoxic enzymes from primary granules of neutrophils at infection sites harms host cells, causing severe inflammation [30,50]. In addition, excess secretion of these enzymes is related to various inflammatory disorders, such as severe episodes of asthma, acute lung injury, rheumatoid arthritis, and some granulomatoses [30,51]. To investigate the impact of Hv1/VSOP deficiency, Hv1/VSOP-deficient mice were challenged with the opportunistic pathogen *Candida Albicans* [29], which is sensitive to ROS [52]. Hv1/VSOP-deficient mice eliminated *C. Albicans* normally when inoculated intranasally, but these mice exhibited more severe inflammation in the lung than wild-type mice [29]. The lung inflammation in Hv1/VSOP-deficient mice was typically pyogranulomatous, characterized by aggregates of large foamy macrophages, lymphocytes and neutrophils [29]. These results indicate that Hv1/VSOP suppresses inflammation through the inhibition of excess degranulation of primary granules upon pathogen infection.

## 4. Regulation of Neutrophil Migration by Hv1/VSOP

### 4.1. Hv1/VSOP Regulation of Ca^2+^-Induced Random Movement of Neutrophils

Neutrophils are highly locomotive immune cells. These cells quickly reach infection sites upon infection following several guidance cues and then resolve the infection by engulfing and digesting pathogens through the release of degradative enzymes and ROS. Directional migration of neutrophils is regulated by Ca^2+^ and cAMP [53] and by several intracellular signals, including AKT/PKB [54] and MAPKs [55,56].

**Figure 3:**
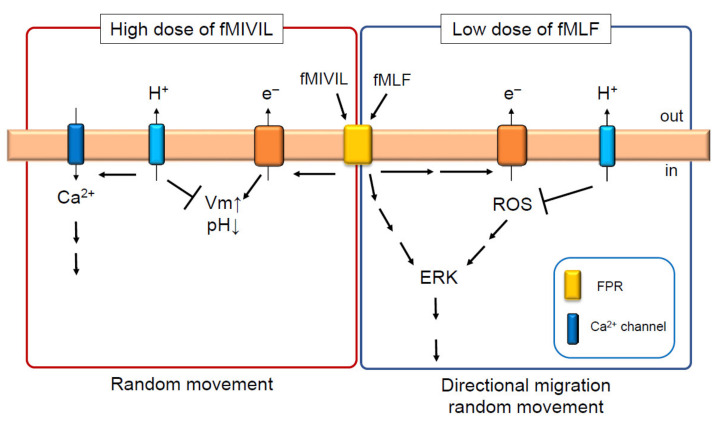
Hv1/VSOP controls the migration of neutrophils by positively and negatively regulating ROS production. In the presence of high dose of fMIVIL, Hv1/VSOP promotes Ca^2+^ influx at the plasma membrane by suppressing depolarization induced by NAPDH oxidase [22]. This is necessary for the proper movement of neutrophils in response to fMIVIL [22]. In the presence of low doses of fMLF, Hv1/VSOP inhibits ROS production [25], leading to the suppression of extracellular signal-regulated kinase (ERK) activity and migration of neutrophils in response to fMLF [25]. Arrow and T arrow indicate promotion and inhibition, respectively, for Vm, pH, Ca^2+^, ROS and so on.

In unexcitable cells, Ca^2+^ influx from the plasma membrane is diminished by depolarization because of the reduced Ca^2+^ driving force. Hv1/VSOP on the plasma membrane inhibits depolarization caused by activation of NAPDH oxidase; therefore, it is possible that Hv1/VSOP regulates Ca^2+^ influx. Indeed, Ca^2+^ influx was diminished in Hv1/VSOP-deficient neutrophils compared with wild-type neutrophils when stimulated with PMA under conditions of Ca^2+^ store depletion [22], indicating reduced Ca^2+^ influx at the plasma membrane. Reduced [Ca^2+^]_in_ was also observed in Hv1/VSOP-deficient neutrophils by stimulation with *N*-formyl-Met-Ile-Val-Ile-Leu (fMIVIL) [22], a chemoattractant and potent agonist of formyl peptide receptor (FPR). FPR is a G-protein coupled receptor and its activation stimulates the increase in [Ca^2+^]_in_ and cAMP, and various signal cascades [57,58]. Interestingly, Hv1/VSOP-deficient neutrophils exhibited a defect in random movement in the presence of fMIVIL [22]. These cells move more slowly and migrate a small distance. The migration defect in Hv1/VSOP-deficient neutrophils was fully restored in the presence of a Ca^2+^ ionophore [22]. Hv1/VSOP, therefore, has a role in promoting neutrophil migration by regulating Ca^2+^ influx (Figure 3).

A phenotype similar to that found in mouse neutrophils was also observed in zebrafish neutrophils in an in vitro study [28]. Ca^2+^ influx was diminished in Hv1/VSOP-deficient neutrophils isolated from zebrafish blood when stimulated with the same concentration of fMIVIL as used on mouse neutrophils, indicating the conserved function of Hv1/VSOP across vertebrate animals. Real-time behavior of neutrophils was also investigated in living zebrafish in response to tissue injury and acute bacterial infection. There was no clear difference in neutrophil migration in response to the above treatment between wild-type and Hv1/VSOP-deficient zebrafish neutrophils. At injury and infection sites, several kinds of danger signals, such as ATP and cytokines, are released from neighboring cells, which may help Hv1/VSOP-deficient neutrophil migration to those sites.

### 4.2. Hv1/VSOP Regulation of ERK-Induced Directional and Random Migration of Neutrophils

Another aspect of Hv1VSOP function was revealed from the analysis of directional migration of mouse neutrophils [25]. Surprisingly, Hv1/VSOP has an inhibitory effect on neutrophil migration in response to low N-formyl-Met-Leu-Phe (fMLF) doses. fMLF is another FPR agonist. Hv1/VSOP-deficient neutrophils exhibited enhanced migration in response to fMLF at less than 1 µM, while neutrophils normally responded to fMLF at more than 10 µM [25]. Of note, Hv1/VSOP-deficient neutrophils responded to lower doses of fMLF, such as 125 nM, a concentration at which wild-type neutrophils rarely respond [25]. These cells also exhibited enhanced random migration with the same range of fMLF doses as in directional migration [25]. More surprisingly, the amount of ROS released from Hv1/VSOP-deficient neutrophils was greater than in wild-type cells when these cells were stimulated with 1 µM fMLF [25]. Furthermore, basal ROS production before fMLF stimulation was also increased in Hv1/VSOP-deficient neutrophils [25]. The enhanced response to low dose fMLF was suppressed by inhibiting NADPH oxidase activity with DPI [25], indicating that the enhanced response to fMLF is caused by increased ROS. These results indicate that Hv1/VSOP inhibits ROS production to suppress migration in response to low doses of fMLF (Figure 3).

Chemoattractants, such as interleukin 8 (IL8), leukotriene B4 (LTB4), and fMLF are known to stimulate ROS production in neutrophils [58,59], although the extent of ROS production is much smaller than by stimulation with phagocytosis and PMA [46]. However, roles of minor ROS have awaited examination in neutrophils. Two groups have reported that minor ROS regulate directional migration by actin remodeling [60] and receptor internalization of FPR [61]. This regulation is thought to be necessary for halting cell migration at infection sites and increasing the probability of pathogen encounters [60,61]. However, Hv1/VSOP was found to regulate cell migration in a distinct way from these findings. The number of receptors and their sensitivity for FPR was normal [25], excluding the possibility that an increased number and sensitivity of FPRs can facilitate the response to low doses of fMLF. Intracellular Ca^2+^ was also normal in these cells [25], indicating that other mechanisms may be at play. Extracellular signal-regulated kinase (ERK) and p38 MAPKs regulate neutrophil migration, and time-dependent increased activity of MAPKs upon fMLF stimulation has been reported [55,56]. These molecules are also indirectly regulated by ROS [62,63]. We found that the extent of ERK activation is altered in Hv1/VSOP-deficient neutrophils compared with wild-type neutrophils: while a time-dependent, modest activation of ERK was seen in wild-type neutrophils, a remarkably greater and prolonged activation was observed in Hv1/VSOP-deficient neutrophils [25]. In contrast, there was no clear difference between these genotypes in the activation of p38 [25]. Inhibiting ROS production by DPI completely suppressed the enhanced activation of ERK in both duration and amplitude [25]. Collectively, the enhanced migration of Hv1/VSOP-deficient neutrophils is caused by the following mechanism: increased ROS in Hv1/VSOP-deficient neutrophils causes prolonged and increased activation of ERK, leading to enhanced migration in response to low doses of fMLF.

How does Hv1/VSOP inhibit ROS production? The mechanism is still unclear, but Hv1/VSOP might inhibit ROS production through the regulation of actin remodeling. In Hv1/VSOP-deficient neutrophils, a thicker F-actin ring was observed surrounding phagosomes [22]. An increased proportion of F-actin was also reported in microglia, another type of phagocyte. Cytoplasmic F-actin was more abundant in Hv1/VSOP-deficient microglia than in wild-type microglia [20]. Interestingly, in wild-type microglia, p67, a subunit of NADPH oxidase, colocalized with F-actin, but the proportion of p67 colocalizing with F-actin was diminished in Hv1/VSOP-deficient microglia [20]. Moreover, Hv1/VSOP-deficient microglia produced larger amounts of ROS than wild-type microglia when stimulated with PMA. These results indicate that the increase in actin-unbound fraction of p67 leads to the increased full complex of NADPH oxidase, resulting in higher ROS production. Actin remodeling by Hv1/VSOP is probably involved in the inhibition of ROS in neutrophils.

## 5. Conclusions and Future Perspectives

**Figure 4:**
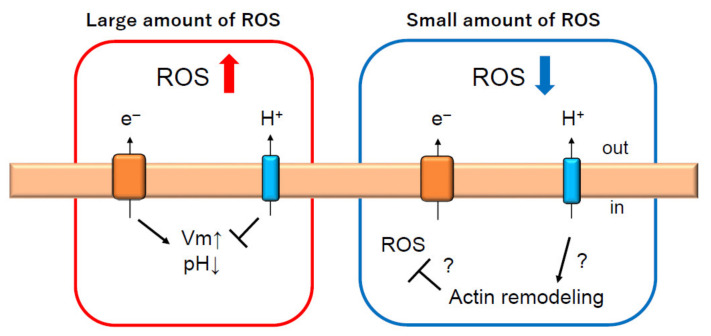
Hv1/VSOP is two-way player regulating ROS production in neutrophils. Under conditions of phagocytosis, which requires a large production of ROS, Hv1/VSOP promotes ROS production to compensate charge imbalance induced by NADPH oxidase activation [12,22,26,28]. However, under conditions when a small amount of ROS is produced, Hv1/VSOP inhibits ROS production [25], which might be regulated through actin remodeling [20,22]. Arrow and T arrow indicate promotion and inhibition, respectively, for Vm, pH, ROS and actin remodeling. Red and blue arrow indicate increase and decrease of ROS production, respectively.

We have presented Hv1/VSOP as a two-way player in neutrophils (Figure 4). Hv1/VSOP not only promotes but also inhibits ROS production. It appears that mechanisms inhibiting ROS production by Hv1/VSOP are distinct from the conventional function of this channel, in that Hv1/VSOP regulates membrane potential and pH, which change with the activation of NADPH oxidase. Although it is still not clear how Hv1/VSOP inhibits ROS production, regulation of actin remodeling by Hv1/VSOP must be a clue to solve this function. Hv1/VSOP localizes to intracellular vesicles in neutrophils [29] and microglia [20], but the function of Hv1/VSOP on endomembranes has not been determined. Recent findings in plasmacytoid dendritic cells shows that Hv1/VSOP acts on endosomes to regulate ROS production and endosomal pH [21]. Hv1/VSOP on endomembranes, including endosomes, might control the activity and the number of NADPH oxidase complexes through the regulation of actin remodeling. Future studies will uncover mechanisms that inhibit ROS production by Hv1/VSOP and make it possible to understand how Hv1/VSOP acts as a two-way player in the regulation of ROS production in neutrophils. Understanding the functions of Hv1/VSOP will help in the development of treatments for several diseases related to neutrophil function.

## Figures and Tables

**Table 1:** Summary of Hv1/VSOP functions in neutrophils.

Cellular Function	Effect ^1^	Stimulus	Reference
Depolarization	inhibition	PMA	[22]
Acidification of cytoplasm	inhibition	PMA, Zymosan	[22,23,24]
Ca^2+^ influx from plasma membrane	promotion	PMA, fMIVIL	[22]
ERK signal	inhibition	fMLF	[25]
O_2_^−^ and H_2_O_2_ production	promotion	PMA, Zymosan, fMIVIL	[12,19,22,26,27,28]
	inhibition	fMLF	[25]
HOCl production	inhibition	PMA	[29]
Bacterial killing	promotion	*Staphylococcus aureus*	[30]
Migration	promotion	fMIVIL	[22]
	inhibition	fMLF	[25]
Degranulation	inhibition	PMA, IgG	[29]

^1^ “Effect” indicates the function of Hv1/VSOP for cellular functions.

## Abbreviations

DPI	Diphenyleneiodonium
ERK	Extracellular signal-regulated kinase
fMIVIL	N-formyl-Met-Ile-Val-Ile-Leu
fMLF	N-formyl-Met-Leu-Phe
FPR	Formyl-peptide receptor
NADPH	Nicotinamide Adenine Dinucleotide Phosphate
VSOP	Voltage-sensor domain-only protein
MPO	Myeloperoxidase
O_2_^−^	Superoxide anion
PMA	Phorbol 12-myristate 13-acetate
BCECF	2′,7′-bis(2-carboxyethyl)-5,6-carboxyfluorescein
DiBAC_4_(3)	Bis-(1,3-Dibutylbarbituric Acid)-Trimethine Oxonol
ROS	Reactive oxygen species
